# Supplementary material for: The coupling of the hydrated proton to its first solvation shell
Source: Nat Commun. 2022 Oct 18;13:6170. doi: 10.1038/s41467-022-33650-w (PMC9579203; doi:10.1038/s41467-022-33650-w)
Supplement: Supplementary file 1 — Supplementary information [file 41467_2022_33650_MOESM1_ESM.pdf]

Supplementary information

The coupling of the hydrated proton to its first solvation shell

Markus Schröder*, Fabien Gatti, David Lauvergnat,

Hans-Dieter Meyer, Oriol Vendrell*

September 25, 2022

*Correspondence: markus.schroeder@pci.uni-heidelberg.de; oriol.vendrell@uni-heidelberg.de

This PDF file includes:

Supplementary text

Supplementary Figures 1 to 10

Supplementary Tables 1 to 4

Supplementary References

Supplementary Note 1: IR spectrum of the Eigen cation

Supplementary Figure 1 shows the calculated absorption spectrum of the Eigen cation ($H_9O_4^+$) in comparison with the experimental spectra from Refs. 1 and 2. The overall agreement of calculated and experimental spectra is very good.

The calculated spectrum contains a global shift of 70 cm^{-1} (which on average accounts to about 2 cm^{-1} per mode) towards lower energies to better match the main measured bands. The shift is a consequence of the fact that the propagated wavefunction is much more structured and hence is less converged than the wavefunction used for calculating the ground state reference energy. Note that there is no shift for reduced dimensionality (14D) calculations.

The resolution of the calculated spectrum is approximately 30 cm^{-1} and limited by the 1 picosecond duration of the dipole-dipole correlation function. Longer correlation times require more tightly converged wavefunctions, which become prohibitive computationally on current hardware for such a large system. As a consequence, the peak positions are considered accurate to a few tens of cm^{-1} and are rounded to 10 cm^{-1} when listed or discussed. The calculated peak positions are listed in Table 1 alongside with experimental results and assignments.

Supplementary Figure 1 and Supplementary Table 1 indicate the assignments of the main peaks, where we adhere to the labeling convention in Ref. 1. The peaks labelled on top of the experimental spectra have been discussed in Refs. 1, 2, 4–7, whereas peaks labelled on top of the calculated spectrum correspond to new assignments. The quantum mechanical localized excitations indicated in the assignments represent the largest contribution to the corresponding eigenstate. The assignment is established through cross-correlating locally excited test states with the propagated, dipole-operated ground state. Based on the cross-correlation analysis, the band a_{11} can be unambigously assigned to an overtone of the hydronium wagging. Previous works had suggested a similar asignment without detailed reference to specific overtone and

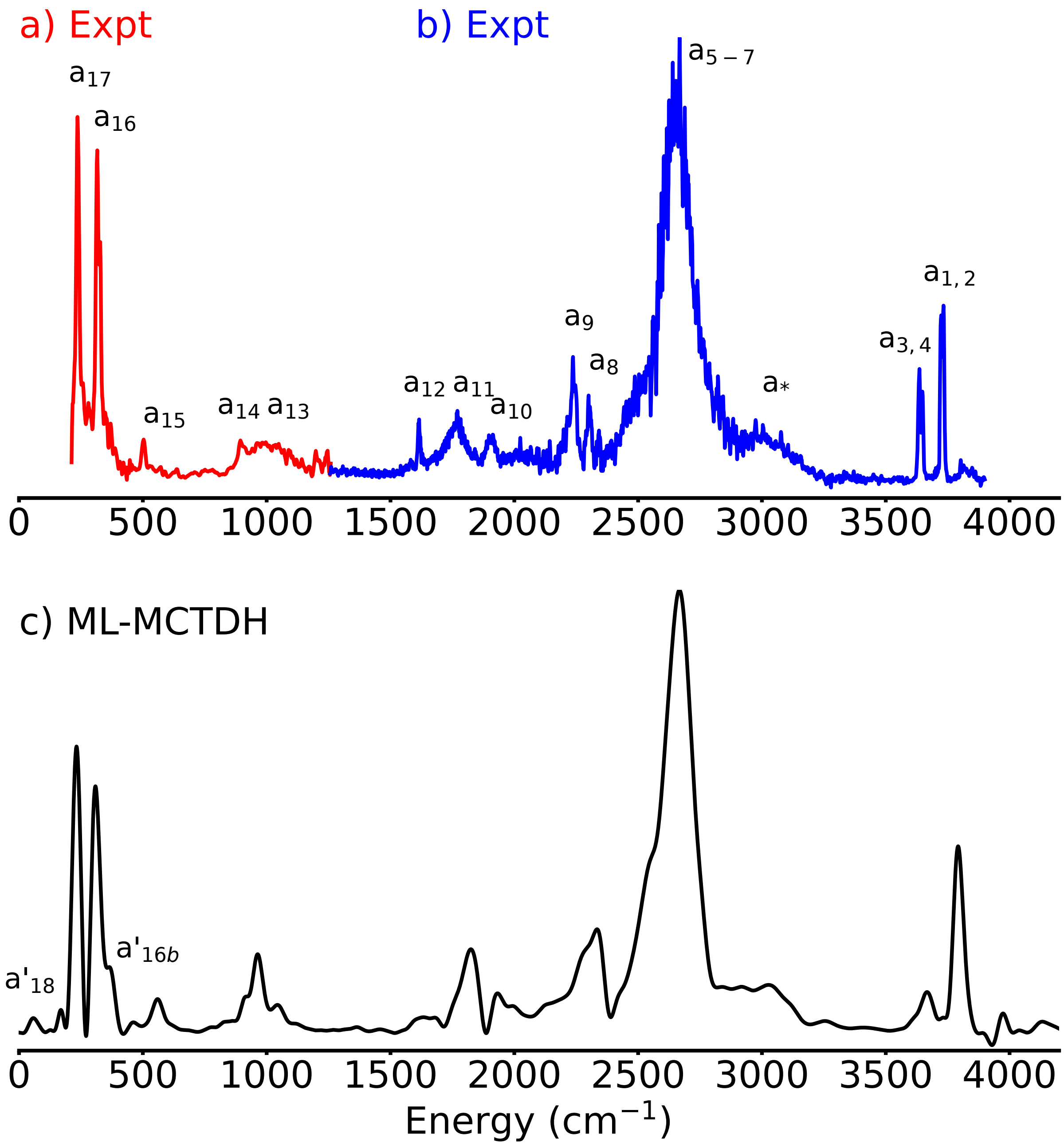

Supplementary Figure 1: Absorption spectrum of the Eigen Cation $H_9O_4^+$. a) Experimental spectrum from Ref. 2, b) Experimental spectrum from Ref. 1, c) Calculated spectrum (red-shifted 70 cm^{-1} to match experimental line positions). Source data are provided under `https://doi.org/10.5281/zenodo.7064870`.

Supplementary Table 1: Peak positions, labels and assignments. Assignments of primed labels this work, other assignments from Refs. 1, 2, 4, 5. In column 2, we state most important zero order assignments with number of quanta and symmetry in the three arms (ABC), where (+++) represents $|100\rangle + |010\rangle + |001\rangle$, (-++) represents $-2\,|100\rangle + |010\rangle + |001\rangle$ and (0+-) represents $|010\rangle - |001\rangle$. (-++) and (0+-) are degenerate in the S3 permutation symmetry group. The coordinates, second column, are explained below. All energies in cm^{-1}. The MCTDH results include a red-shift of 70 cm^{-1} to match peaks a_{16} and a_{17}. *) from cross-corrlation analysis.

			Peak positions		
Assignment		Label	Expt[2]	Expt[1]	MCTDH
Global O_4 pyramidalization	$\theta_{1,(+++)}$	a'_{18}			60
Water ligand wagging	$\lambda_{1,(-++)}$	a_{17}	236		240
O-O stretching	$R_{1,(-++)}$	a_{16}	316		310
Water ligand rocking	$\beta_{1,(+++)}$	a'_{16}			370
Water ligand wagging overtone	$\lambda_{2,(-++)}$	a_{15}	503		560
Hydronium wagging	$y_{1,(-++)}$	a_{14}	894		920
Hydronium umbrella	$y_{1,(+++)}$	a_{13}	1014		970
OH-bend water ligands	$b_{1,(-++)}$	a_{12}	1615	1615	1620^{*}
Hydronium wagging overtone	$y_{2,(-++)}$	a_{11}	1760	1770	1830
Hydronium umbrella + wagging		a_{10}		1909	1940
OH-bend + frustrated hydronium rotation		a_9		2237	2290
OH-bend + hydronium umbrella		a_8		2300	2340
Hydronium O-H stretch	$z_{1,(-++)}$	a_{5-7}		2653	2670
Symmetric OH- stretch water ligands	$v^{(s)}_{1,(-++)}$	$a_{3,4}$		3636	3670
Asymmetric OH-stretch water ligands	$v^{(a)}_{1,(-++)}$	$a_{1,2}$		3724	3800

combination band.[4]

Our analysis provides further assignments including peaks a_1 – a_{5-7}, and a_{13} – a_{17} (see cross-correlation analysis below). The peak a_{12} at 1615 cm^{-1} in the experiment which belongs to O-H bending is blurred in the calculated spectrum but is found in the cross-correlation analysis at 1620 cm^{-1} (see cross-correlation analysis below). We have confirmed, based on separate calculations using a different multilayer tree, that this blurring is due to a lack of convergence of the propagated wavefunction. The subtree of the hydronium core is especially challenging to converge and highlights the very strong coupling of the central unit with its solvation shell. This very strong coupling results in possibly the least well understood feature of the gas-phase

spectrum of the Eigen cation, namely the very broad absorption band centered at 2600 cm^{-1} in Supplementary Fig. 1.

Similarly to the much better understood Fermi-resonance double peak in the Zundel cation,[8,9] this very broad band has its origin in the coupling of the hydronium O-H stretch modes in the Eigen cation to other vibrational modes of the system.[1] The agreement of the width and shape of this band between the calculated and experimental spectra is excellent. The spectral resolution of the calculation is about 10 times narrower than the calculated band's width, indicating that this is a genuine characteristic of the band and not caused by experimental artifacts, e.g., due to the tagging agent. The pre-band features a_{11} – a_8 and post-band feature a_* are all reproduced as well.

The localized excitation of the hydronium O-H stretch coordinates of the cation with a model dipole operator $\mu_{\text{test}} = -2z_{\text{A}} + z_{\text{B}} + z_{\text{C}}$ results in a spectrum with all fundamental spectral features of the central proton modes. This operator has the same symmetry as the z-component of the dipole operator, but it exclusively operates on the three hydronium O-H stretch coordinates $z_{\text{X=A,B,C}}$. The coordinate z_{X} describes the proton motion parallel to the corresponding O-O vector. The coordinate system is explained in Supplementary Figures 8 and 9. As seen in Supplementary Fig. 2 (blue curve), the bands related to the proton motion span about 1500 cm^{-1}, and the main central O-H stretch feature is nearly identical to the one in the full spectrum (black curve). The character of the various peaks flanking the broad O-H stretch band is listed in Table 1 and comprises excitations of the hydronium core, oxygen-oxygen stretches and water bending motions in the first solvation shell.

Using reduced dimensional models we explain the pronounced broadening of the O-H stretch peak by coupling with many other states. Combinations of low excitations in the hydronium core and excited states in O-O - distance and ligand wagging modes play an important role in this broadening process. The involvement of wagging states and O-O distance can be

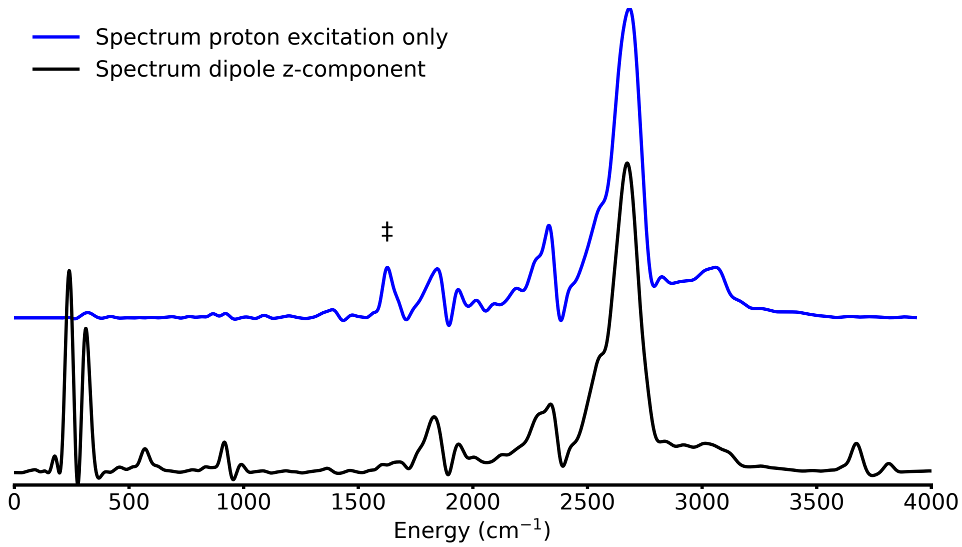

Supplementary Figure 2: Spectrum obtained with a model dipole exclusively operating on proton transfer coordinates (blue curve) in comparison with the spectrum of the full dipole z-component (black curve). Excitation of the hydronium O-H stretch modes excites multiple other states and leads to a characteristically broad signal. The peak marked with ‡ at 1620 cm^{-1}, blurred in the dipole-spectrum, corresponds to O-H bending of the ligand water molecules.

seen as an analogy to the Zundel cation, where a Fermi resonance between one excitation of the proton transfer and a combination mode of the O-O distance mode and a wagging overtone exists. As opposed to the Zundel cation, where only one combination excitation broadens/splits the proton transfer line, there are many in the Eigen cation.

Supplementary Note 1.1: Spectral components

Supplementary Fig. 3 shows the averaged calculated spectrum (black curve) and the spectra obtained with the dipole constituents μ_x (red curve), μ_y (green curve) and μ_z (blue curve) for H_9O_4. The single spectra are to scale, i.e., the single dipole component spectra are displayed according to their contribution to the averaged spectrum.

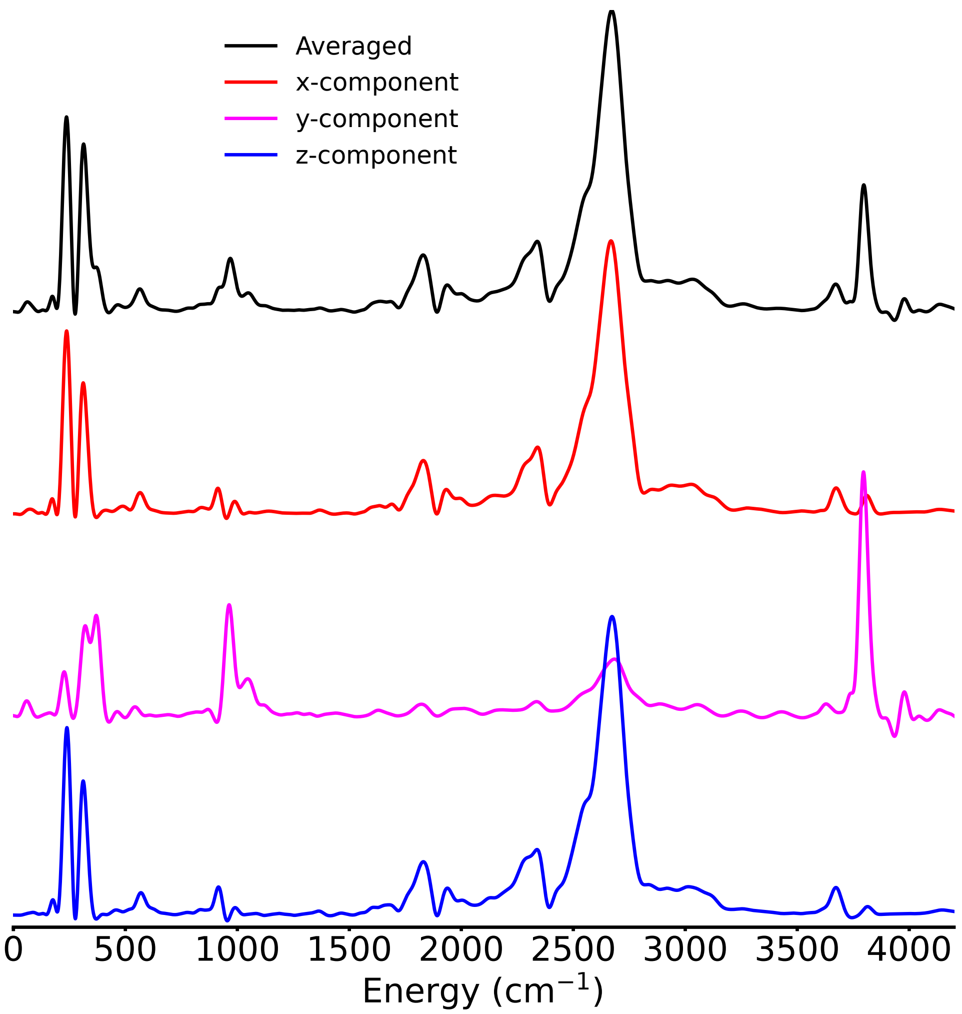

Supplementary Figure 3: Calculated averaged absorption spectrum (upper panel) and $x-$, $y-$ and $z-$ components (lower panel) of the Eigen cation, $H_9O_4^+$. Relative intensities of the components according to their contribution to the averaged spectrum. Source data are provided under `https://doi.org/10.5281/zenodo.7064870`.

Supplementary Note 2: Assignments

We restrict our discussion below to the cases $i = z$ (in-plane, parallel to one hydrogen bond of the molecule) and $i = y$ (out-of-plane) as the states for $i = x$ (remaining in-plane) resemble the features of the other in-plane dipole component $i = z$.

In Supplementary Fig. 4 the Fourier transforms of the cross-correlations of test states with the time-propagated dipole-operated ground state for the z-component of the dipole are given in panels b-k) while panel a) shows the Fourier transformed auto-correlation function (scaled by factor 4 above 500 cm^{-1}). The peak heights of the auto-spectrum hence correspond to the population of the respective eigenstates in the dipole-operated ground state. Note that the labels in Supplementary Fig. 4 denote the correlation function while the graphs show their Fourier transform without the ω prefactor, Eq. (5) of Methods. The curves are color-coded for different liner combinations of coordinate operators: black (+++), red (-++) and blue (0+-).

In panels b-e) the cross-correlations for the b) global O_4 umbrella motion, c) the asymmetric wagging of the outer water molecules, the d) asymmetric O-O stretch and e) the asymmetric double excited wagging signals are shown, respectively. Other liner combinations than the one displayed have not been found to have significant intensity.

In panel f) the symmetric umbrella (black) and asymmetric out-of plane wagging (red) of the central hydronium H-atoms are probed. The two curves are to scale, i.e., the relative peak height corresponds to the relative intensity of both signals.

The ligand water O-H-bending signal, which is blurred in the calculated spectrum of H_9O_4, can be clearly identified in the cross correlation analysis, panel g). The bending is modeled as a linear combination of the internal Jacobi vectors of the waters. We believe that the blurring is due to the relatively small basis set used here.

Panel h) of Supplementary Fig. 4 shows the cross-correlation signal of the doubly excited

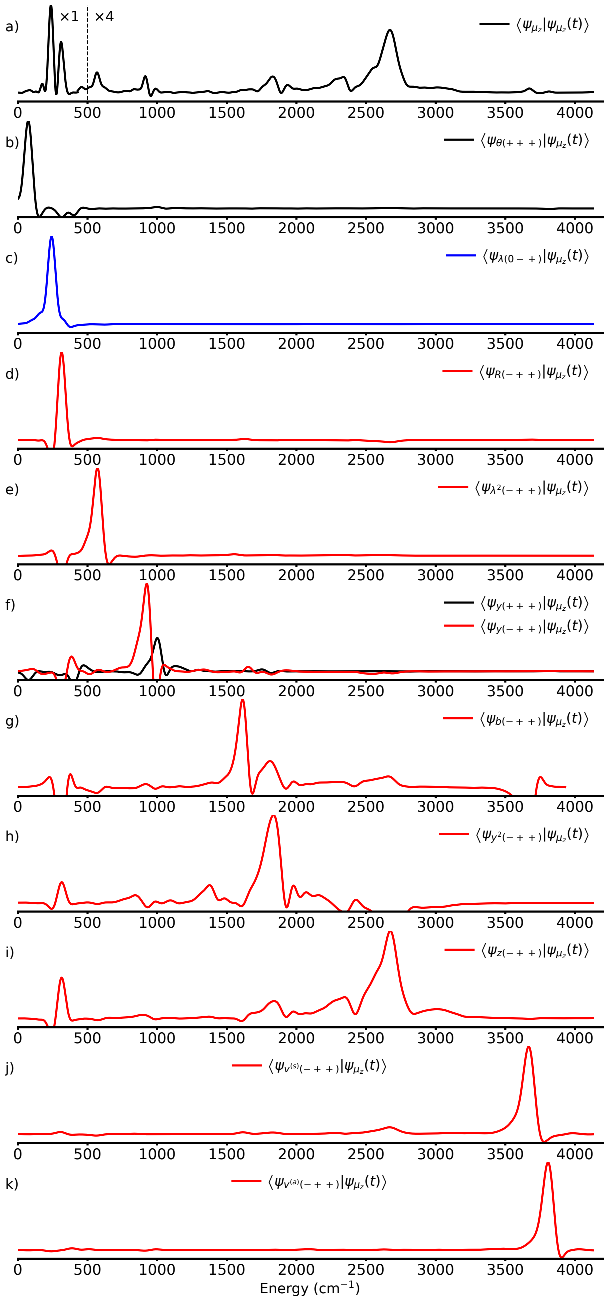

Supplementary Figure 4: Fourier transforms of cross-correlation functions between the time-dependent μ_z-operated ground state of $H_9O_4^+$ and test states. The labels show the definition of the correlation function. The curves are scaled to a maximum value of unity. Where more than one curve is shown in one panel their relative weights are to scale. Panel a) shows the Fourier transform of the auto-correlation function scaled by a factor of four above 600 cm^{-1}.

asymmetric umbrella motion of the central hydronium H-atoms. The main contribution of this signal lies slightly below 2000 cm^{-1} matching the respective peak in the absorption spectrum.

In panel i) the asymmetric O-H of the central hydronium are probed. One clearly sees that the broad feature in at 2700 cm^{-1} corresponds to the hydronium O-H stretch which is correlated with many other states in particular between 1600 and 3500 cm^{-1}. Also the O-O distance state, which appears near 450 cm^{-1} gains significant intensity.

Finally in panel j) and k) the symmetric and asymmetric O-H-stretching of the outer water ligands are probed. For the symmetric O-H-stretching a linear combination of the internal Jacobi vectors of the water ligands has been used.

Furthermore, for the remaining states of the hydronium core above 2000 cm^{-1}, it proved difficult to create appropriate test states such that these signals could not be unambiguously assigned.

In Supplementary Fig. 5, the Fourier transforms of the cross-correlations of test states with the time-propagated dipole-operated ground state for the y-component of the dipole are given. Panel a) again shows the auto-correlation signal of the μ_z-operated ground state. The signal is enlarged by a factor of two above 1500 cm^{-1}. The structure in the lower frequency range up to 1000 cm^{-1} is much more pronounced than for the z-component. Panels b-f) show the respective cross-correlation signals of the b) O_4 global angles, c) wagging of the outer water ligands, d) O-O stretch, e) H_2O ligand rocking, and f) asymmetric wagging overtone.

In panel g) the asymmetric wagging (black curve) and symmetric umbrella (red curve) signals of the central hydronium are shown. The feature at 1000 cm^{-1} is mainly caused by the umbrella motion and a second peak on the high energy shoulder of the umbrella motion is visible.

Panel h) shows the cross-correlation signals with the symmetric and asymmetric O-H-stretching modes of the central H_3O^+. Both peaks contribute approximately equally to the

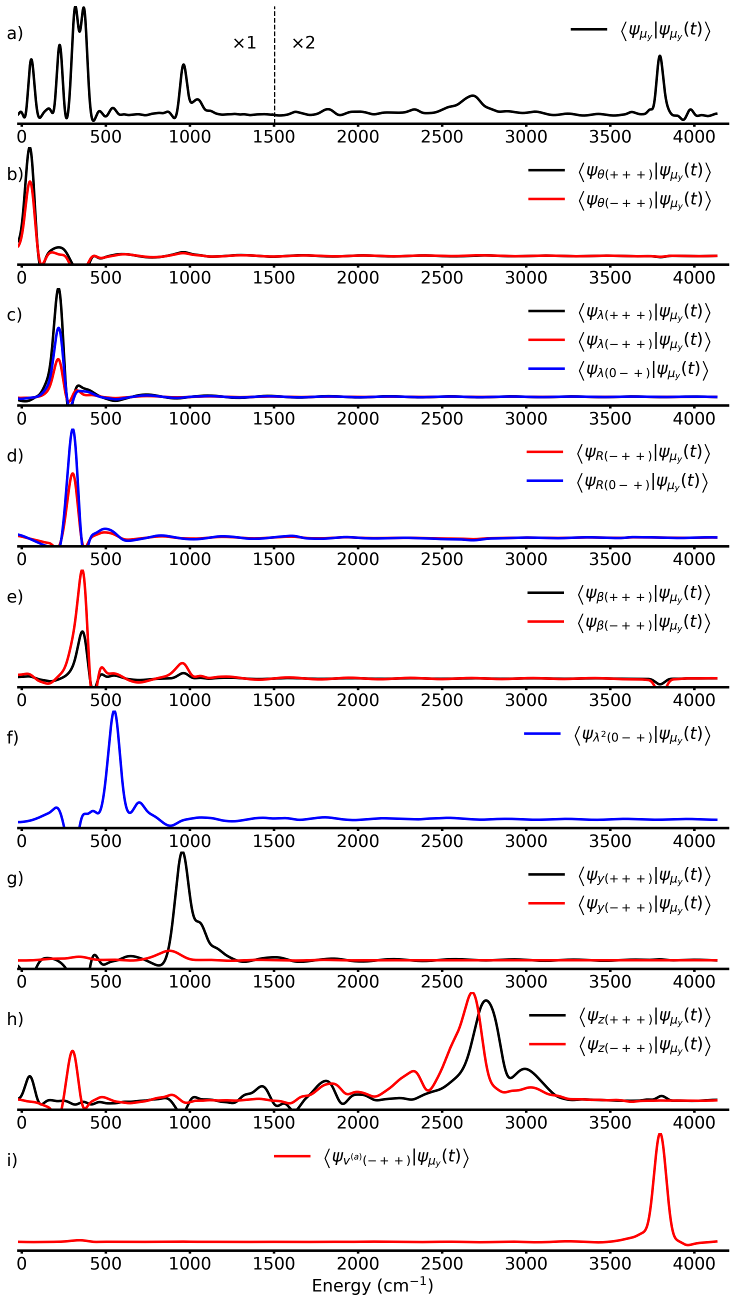

Supplementary Figure 5: Fourier transforms of cross-correlation functions between the time-dependent μ_y-operated ground state of $H_9O_4^+$ and test states (details see text) to probe existence of a corresponding state. The curves are scaled at maximum to unity. Where more than one curve is shown in one panel their relative weights are to scale. The upper panel shows the Fourier transform of the auto-correlation function scaled by a factor of two above 1500 cm^{-1}.

signal in the y-component of the dipole. Note, that the peaks are energetically quite separated but equally broad. Excitation of the hydronium O-H stretch hence seems to co-excite quite different states in the energetic vicinity of the original excitation. Note that in case of the symmetric O-H-stretching also the global O_4 scaffold is affected. The asymmetric O-H-stretching of the outer water ligands is finally probed in panel i).

Supplementary Note 3: Transition from $H_5O_2^+$@Eigen to Zundel

To justify the argumentation of the $H_5O_2^+$ subunit being the fundamental dynamical building block to understand the spectrum of the Eigen cation we calculated spectra of various configurations as depicted in Supplementary Fig. 6. It illustrates the influence of the presence of the static Eigen environment on the position of O-H stretch peak.

Supplementary Fig. 6 a) shows the spectrum obtained with the dipole moment z-component using a full-dimensianal quantum mechanical model with the hydronium O-H stretch peak visible at 2700 cm^{-1}. In Supplementary Fig. 6 b) only one $H_5O_2^+$ subunit of the total Eigen cation is modeled dynamically while all other coordinates are frozen to their equilibrium position. One observes that the spectrum changes slightly, but the broad O-H stretch peak remains approximately at 2700 cm^{-1}.

It has been shown before that the position of the O-H stetch peak strongly depends on the O-O distances in protonated water clusters[11–15] In Supplementary Fig. 6 c) therefore, the two frozen water molecules are moved away from the dynamical $H_5O_2^+$ subunit by increasing the respective O-O distance by 0.55 Å to 3.12 Å. This has dramatic consequences for the spectrum as the O-H stretch peak is now red-shifted by about 900 cm^{-1}.

The PES of Refs. 13, 16 does not facilitate larger O-O distances hence Supplementary Fig.

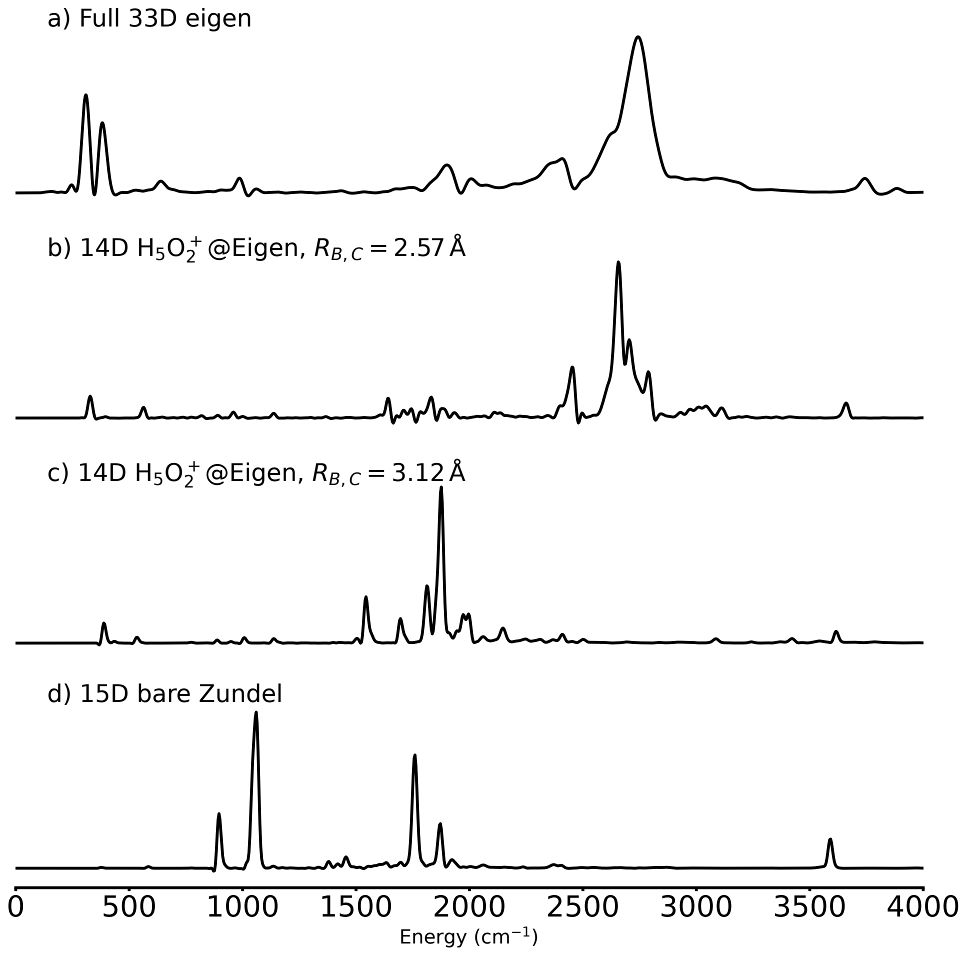

Supplementary Figure 6: Spectra obtained with the z-component of the dipole moment surfaces for various systens: a) Full 33D calculation of the eigen cation b) 14D calculation of the $H_5O_2^+$ subunit as in Supplementary Fig. 4a) of the main text with the two frozen O-O distances set to equilibrium positions of 2.57 Å. c) Same as in b) but with the two frozen O-O distances set to 3.12 Å. d) Full 15D Zundel using the PES from Ref. 10.

6 d) shows the spectrum of the bare Zundel cation for comparison. This spectrum has been obtained using the PES and DMS of Huang *et al.*[10] The O-H stretch peak here is located at slightly above 1000 cm^{-1}, hence red-shifted by about 1600 cm^{-1} compared to Eigen.

Kinetic energy operator

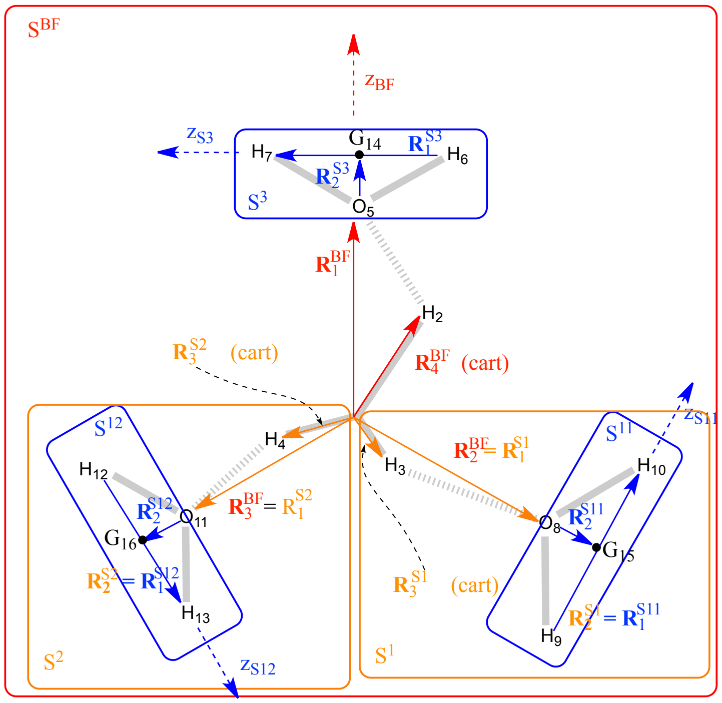

Supplementary Figure 7: Structure of the vectors for the description in polyspherical coordinates. Two Jacobi vectors (one from one H atom to the other and the second from the O atom to the middle of H_2) are used to describe the molecules of water (in blue).

Coordinates and primitive basis

Table 2 outlines the physical coordinates, coordinate ranges and number of primitive grid points used for each coordinate. A graphical illustration of the coordinates is also given in Supplementary Fig. 9.

Potential energy and DMS fits

The potential energy and dipole moment surface fits have been created with in total $7 \cdot 10^6$ sampling points which were obtained with a Metropolis algorithm at different temperatures

Supplementary Table 2: Primitive coordinates and grids.

Coordinate label	physical meaning	DVR type	No. Points	range	
θ	O_4 pyramidalization	sin	11	2.142 – 3.142	rad
$\cos(\varphi_{\mathrm{AB}})$, $\cos(\varphi_{\mathrm{AC}})$	O_4 bending	sin	11	-0.71 – -0.1	
R_{A}, R_{B}, R_{C}	O-O stretching	ho	13	4.3 – 5.9	au
λ_{A}, λ_{B}, λ_{C}	ligand wagging	sin	13	2.169 – 4.114	rad
$\cos(\beta_{\mathrm{A}})$, $\cos(\beta_{\mathrm{B}})$, $\cos(\beta_{\mathrm{C}})$	ligand rocking	sin	9	-0.5 – 0.5	
α_{A}, α_{B}, α_{C}	water rotation around O-O axis	sin	9	-0.5 – 0.5	rad
$r_{1,\mathrm{A}}$, $r_{1,\mathrm{B}}$, $r_{1,\mathrm{C}}$	water H-H distance	ho	9	2.3 – 3.6	au
$r_{2,\mathrm{A}}$, $r_{2,\mathrm{B}}$, $r_{2,\mathrm{C}}$	water (H-H)-O distance	ho	7	0.7 – 1.6	au
$\cos(\nu_{\mathrm{A}})$, $\cos(\nu_{\mathrm{B}})$, $\cos(\nu_{\mathrm{C}})$	water Jacobi angle	ho	7	-0.3 – 0.3	
x_{A}, x_{B}, x_{C}	hydronium H in-plane	ho	9	-0.84 – 0.84	au
y_{A}, y_{B}, y_{C}	hydronium H out-of-plane	ho	9	-0.84 – 0.84	au
z_{A}, z_{B}, z_{C}	hydronium O-H-stretching	ho	9	1.35 – 2.65	au

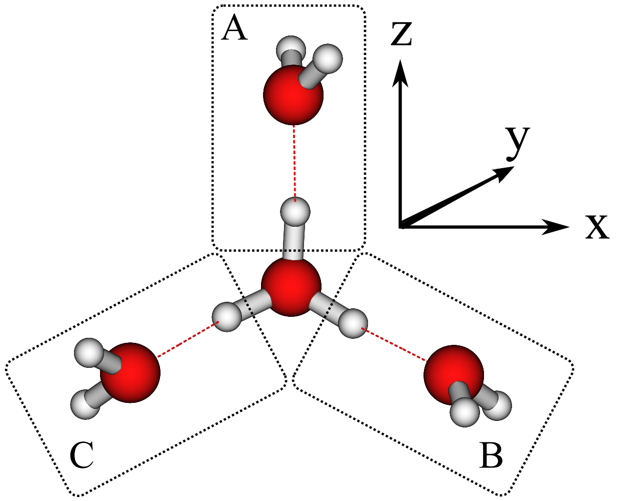

Supplementary Figure 8: Labeling of the three "arms" of the eigen cation as "A", "B" and "C" as well body fixed axis and directions of dipole moment components. The z-component points along the O-O vector of arm "A".

such that the sampling points are distributed according to the weight

$$W(\vec{q}) = \sum_i a_i \exp\left(-\frac{V(\vec{q})}{k_{\mathrm{B}}T_i}\right), \tag{1}$$

where T_i are the different temperatures and k_{B} is the Boltzmann constant. The coefficient a_i is proportional to the number of sampling points used for the given temperature. The number of

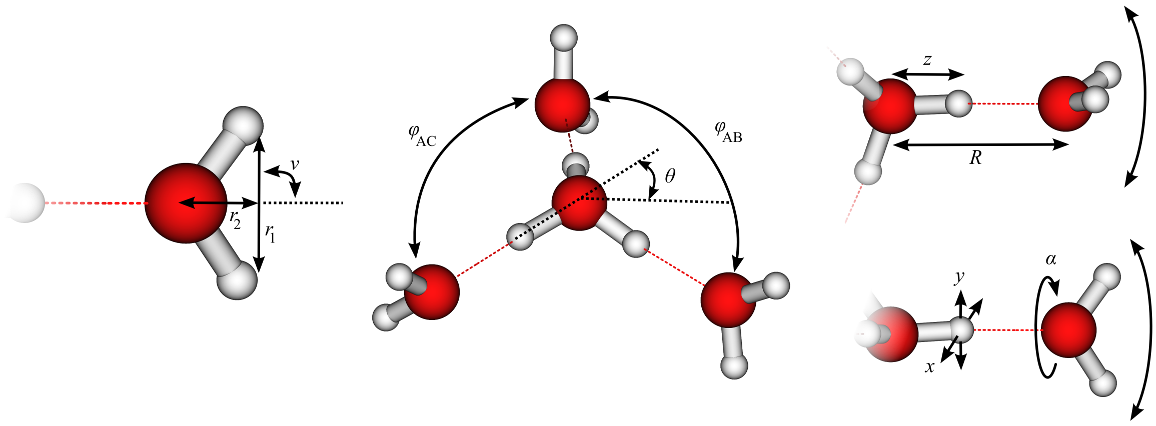

Supplementary Figure 9: Coordinates used for dynamical calculations. Coordinates defined in one "arm" of the cluster are additionally labeled with subscript A, B or C, depending in which arm they are defined. The three arms are labeled clockwise A, B and C.

points for each temperature and the root-mean-square error (RMSE) of the fit on a validation set obtained with the same temperature but of a fixed size of 10^7 points are listed in Table 3.

$k_\mathrm{B}T$ in cm^{-1}	No. Points (creating set)	RMSE in cm^{-1} on validation set (10^7 points each $k_\mathrm{B}T$)
500	$2.0 \cdot 10^6$	43
1000	$2.0 \cdot 10^6$	77
2000	$2.0 \cdot 10^6$	137
3000	$7.5 \cdot 10^5$	191
4000	$2.5 \cdot 10^5$	244

Supplementary Table 3: Number of sampling points used for generating the PES fit at different temperatures as well as root-mean-square error of the final potential on validation sets.

A similar procedure has been applied to decompose the x, y and z components the molecular dipole moment surfaces into a sum-of-products form with 1024 terms. Each DMS was fitted and validated with the same sets of points as used for the potential. The RMSE Errors of the fits are listed in Table 4.

$k_\mathrm{B}T$ in cm^{-1}	RMSE in 10^{-2} Debye on validation set		
	x-component	y-component	z-component
500	0.5	0.6	0.7
1000	1.0	1.0	1.2
2000	1.9	1.7	2.3
3000	2.7	2.0	3.2
4000	3.5	2.4	4.0

Supplementary Table 4: Root-mean-square errors of the DMS fits on validation sets with 10^7 points

Wavefunction representation

Within MCTDH the wavefunction is represented in a hierarchical Tucker format where a layered set of basis functions is used to combine physical into logical coordinates. The ML-representation of wavefunction used in the present case is shown in Supplementary Fig. 10.

Supplementary References

[1] Wolke, C. T. *et al.* Spectroscopic snapshots of the proton-transfer mechanism in water. *Science* **354**, 1131 (2016).

[2] Esser, T. K. *et al.* Deconstructing Prominent Bands in the Terahertz Spectra of $H_7O_3^+$ and $H_9O_4^+$: Intermolecular Modes in Eigen Clusters. *Phys. Chem. Lett.* **9**, 798 (2018).

[3] Schröder, M., Gatti, F., Lauvergnat, D., Meyer, H.-D. & Vendrell, O. Data repository for ”The coupling of the hydrated proton to its first solvation shell”. *Zenodo* (2022). URL https://doi.org/10.5281/zenodo.7064870.

[4] Yu, Q. & Bowman, J. M. Communication: Vscf/vci vibrational spectroscopy of $h_7o_3^+$ and $h_9o_4^+$ using high-level, many-body potential energy surface and dipole moment surfaces. *J. Chem. Phys.* **146**, 121102 (2017).

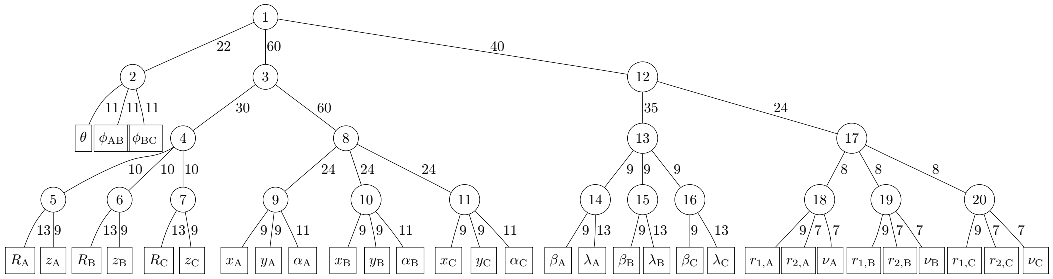

Supplementary Figure 10: Multi-Layer tree used in MCTDH calculations for $H_9O_4^+$. Circles denote vertices of logical coordinates and rectangles of physical coordinates. Edge labels represent numbers of basis functions. Size of wavefunction tensor: $1.2{\cdot}10^6$ complex numbers.

[5] Yu, Q. & Bowman, J. M. High-level quantum calculations of the ir spectra of the eigen, zundel and ring isomers of $h^+(h_2o)_4$ find a single match to experiment. *J. Am. Chem. Soc.* **139**, 10984 (2017).

[6] Duong, C. H. *et al.* Disentangling the complex vibrational spectrum of the protonated water trimer, $H_7O_3^+$, with two-color IR-IR photodissociation of the bare ion and anharmonic VSCF/VCI theory. *J. Chem. Phys. Lett.* **8**, 3782 (2017).

[7] Duong, C. H. *et al.* Tag-Free and Isotopomer-Selective Vibrational Spectroscopy of teh Cryogenically Cooled $H_9O_4^+$ Cation with Two-Color. IR-IR Double Resonance Photoex-

citation: Isolating the Spectral Signature of a single OH Group in the hydronium Ion Core. *J. Phys. Chem. A* **122**, 9275 (2018).

[8] Vendrell, O., Gatti, F. & Meyer, H.-D. Dynamics and infrared spectroscopy of the protonated water dimer. *Angew. Chem. Int. Ed.* **46**, 6918–6921 (2007).

[9] Vendrell, O., Gatti, F. & Meyer, H.-D. Strong isotope effects in the infrared spectrum of the zundel cation. *Angew. Chem. Int. Ed.* **48**, 352 – 355 (2009).

[10] Huang, X., Braams, B. J. & Bowman, J. M. *Ab initio* potential energy and dipole moment surfaces for $H_5O_2^+$. *J. Chem. Phys.* **122**, 044308 (2005).

[11] Fournier, J. A. *et al.* Snapshots of Proton Accommodation at a Microscopic Water Surface: Understanding the Vibrational Spectral Signatures of the Charge Defect in Cryogenically Cooled H(+)(H2O)(n=2-28) Clusters . *J. Phys. Chem. A* **119**, 9425 (2015).

[12] Yu, Q. & Bowman, J. M. How the Zundel ($H_5O_2^+$) Potential Can Be Used to Predict the Proton Stretch and Bend Frequencies of Larger Protonated Water Clusters. *J. Chem. Phys. Lett.* **7**, 5259 (2016).

[13] Heindel, J., Yu, Q., Bowman, J. & Xantheas, S. Benchmark Electronic Structure Calculations for $H_3O^+(H_2O)_n$, n = 0–5 Clusters and Tests of an Existing 1,2,3-body Potential Energy Surface with a New 4-body Correction. *J. Chem. Theory Comput.* **14**, 4553 (2018).

[14] Yu, Q., Carpenter, W. B., Lewis, N. H. C., Tokmakoff, A. & Bowman, J. M. High-Level VSCF/VCI Calculations Decode the Vibrational Spectrum of the Aqueous Proton. *J. Phys. Chem. B* **123**, 7214 (2019).

[15] M, A. B. *et al.* Beyond Badger's Rule: The Origins and Generality of the Structure–Spectra Relationship of Aqueous Hydrogen Bonds. *J. Chem. Phys. Lett.* **10**, 918 (2019).

[16] Qu, C., Yu, Q. & Bowman, J. Permutationally Invariant Potential Energy Surfaces. *Annu. Rev. Phys. Chem.* **69**, 151 (2018).
